# Tribute of Respect to Dr. Holyoke

**Published:** 1828-08

**Authors:** 


6. *Tribute of Respect to Dr. Holyoke.*—We are indebted to the last number of the new *Boston Medical and Surgical Journal* for an interesting account of a tribute of respect, which in this instance at least, is honorable to all the parties concerned. Edward Augustus Holyoke, M. D. & L. L. D., of Salem, Massachusetts, within the present moth, having attained to his hundreth year; a number of the most distinguised physicians of Boston and Salem, determined to give him a public dinner, as an evidence of their veneration for his various excellences of character, displayed thro' three successive generations. The following account of the ceremonies of the day will, we are sure, be read with interest.

"The venerable Nestor of the profession displayed a degree of health and cheerfulness which cannot be called less than wonderful when connected with his extreme age. The firmness and elasticity of his step, in proceeding to and from the Coffee House, where a dinner was prepared for the occasion, though not an unfrequent subject of observation to the community, all of whom look up to him with more than the veneration due to a father, was viewed with a peculiar interest at that time. His rich and graceful dress, also,—of an ante-revolutionary fashion and texture, resembling neitheir in form nor colour the garments of modern time, and of which an adequate conception can only be obtained by the examination of ancient portraits,—deserved and received particular notice. The suavity, ease, and highbred politeness of the old school, displayed by the patriarch in receiving the congratulations of his brethren, and in bidding them adieu at the close of the festival, will long be borne in the minds of those who had the happiness to witness his characteristic exhibition of those qualities.

Dr. Holyoke has so long held a distinguished rank in the profession, that forty-seven years ago, when the Massachusetts Medical Society was organized, he was the first to be elected President of that important and useful institution, to which office he was annually elected for several years; and ten years ago, at the age of ninety, he succeeded the late President Adams as President of the American Academy of Arts and Sciences. The celebration was eminently a medical one, designed to show the respect felt by the profession for him who had so long and so honorably practised it. The only guests from without the profession were the Rev. Dr. Prince and Rev. Mr. Brazer, one long an associate and friend, the other the Pastor of Dr. Holyoke. The company, consisting of about fifty physicians of Boston and Salem, were assembled at the Lafayette Hotel in Salem, when a little before two o'clock Dr. Holyoke entered, accompanied by the committee of arrangements. The gentlemen present were severally introduced to him, and soon proceeded to the dining-room, which was tastefully decorated with flowers by some young ladies, assisted by two medical students. The dinner was well ordered and sumptuous. A blessing was asked by Rev. Dr. Prince, and thanks were offered by Rev. Mr. Brazer. Dr. Jackson, President of the Massachusetts Medical Society, presided at the dinner, assisted by Dr. Oliver of Salem, President of the Essex District Society, as Vice President. The following are some of the toasts.

Our venerable guest;—Venerable for his age; still more so for a life devoted to the interests of humanity and sanctified by religion.

Dr. Holyoke then gave the following:

The Massachusetts Medical Society;—May it flourish and prosper, may it continue to improve the Art for which it was instituted, to the utmost of their wishes, and be the means, under Providence, of alleviating the pains and evils of life, and promoting the happiness of society, by suppressing quackery and rendering the business of the profession as perfect as the nature of things admits. And may each individual of the Society, and every other gentleman here present, enjoy health and prosperity, and the pleasing consciousness that he has contributed somewhat to the advancement and improvement of the public welfare.

This toast was handed to the President in a clear hand writing, having been written by Dr. H. himself without glasses.

Honor to the old age which comes full of honors; to every one that hath shall be given.

The good fellowship of the Medical profession;—In all divisions, let it be our effort to promote an *union by the first intention.*

The Holy-Oak of the American Materia Medica; fructibus decora, sublima attollens ad sidera caput."

Fifteen months ago, we had the honor of a short visit to this venerable centenarian, whom we found disposed and qualified for conversation; and especially inclined to converse on the subject of climate, which had through life been with him a favourite theme. In rising to leave him, he followed us to the door, where we left him making an observation on the state of his thermometer. He is probably the oldest physician living.

